# Degradation of Methylene Blue Dye in the Presence of Visible Light Using SiO_2_@α-Fe_2_O_3_ Nanocomposites Deposited on SnS_2_ Flowers

**DOI:** 10.3390/ma11061030

**Published:** 2018-06-17

**Authors:** Sridharan Balu, Kasimayan Uma, Guan-Ting Pan, Thomas C.-K. Yang, Sayee Kannan Ramaraj

**Affiliations:** 1 Department of Chemical Engineering and biotechnology, National Taipei University of Technology, Taipei 106, Taiwan, bsridharanbsc.12@gmail.com (S.B.); t6679013@gmail.com (G.-T.P.); 2 Precision Analysis and Research Center, National Taipei University of Technology, Taipei 106, Taiwan; 3 PG & Research Department of Chemistry, Thiagarajar College, Madurai 625009, Tamilnadu, India; sayeekannanramaraj@gmail.com

**Keywords:** photocatalyst, flower-like SnS_2_, nanocomposites, visible light, methylene blue

## Abstract

Semiconductor materials have been shown to have good photocatalytic behavior and can be utilized for the photodegradation of organic pollutants. In this work, three-dimensional flower-like SnS_2_ (tin sulfide) was synthesized by a facile hydrothermal method. Core-shell structured SiO_2_@α-Fe_2_O_3_ nanocomposites were then deposited on the top of the SnS_2_ flowers. The as-synthesized nanocomposites were characterized by X-ray diffraction (XRD), Raman spectroscopy, field emission scanning electron microscopy (FE-SEM), transmission electron microscopy (TEM), X-ray photoelectron spectroscopy (XPS), UV–Vis Spectroscopy, Brunauer–Emmett–Teller (BET) surface area analysis, and photoluminescence (PL) spectroscopy. The photocatalytic behavior of the SnS_2_-SiO_2_@α-Fe_2_O_3_ nanocomposites was investigated by observing the degradation of methylene blue (MB). The results show an effective enhancement of photocatalytic activity for the degradation of MB especially for the 15 wt % SiO_2_@α-Fe_2_O_3_ nanocomposites on SnS_2_ flowers.

## 1. Introduction

Environmental pollution is largely caused by the willful disposal of waste and toxic pollutants without proper treatment. Increasingly, this leads to soil and water degradation, which is deleterious to human health [1,2]. In order to rectify this problem, a variety of processes have been adopted to remove pollutants from wastewater, including adsorption [3], oxidation [4], reduction, and flocculation [5]. Among these various methods, photocatalysis has shown good potential for the removal of organic pollutants [6,7,8]. It has been reported that a number of semiconductors can be used to degrade a variety of organic pollutants using irradiation of light with different wavelengths [9,10]. When compared with other organic compounds, dyes are the most tested substrates in photocatalytic degradation under solar light, because dyes are considered as the common industrial pollutants, and their concentrations can be easily monitored using a UV-Vis spectrometer. However, the dyes also absorb light, especially in the visible range, the influence of this photo absorption by dyes should be excluded from the evaluation of the real photocatalytic activity of photocatalysts. Among the various dyes, methylene blue (MB) is a heterocyclic aromatic compound, which is poisonous and highly dangerous and causes breathing hazards, vomiting, hyperhidrosis, and mental disorders [11,12]. Many researchers have focused on thiazine dyes with a dominance of methylene blue, and 37% of published papers reported on the photodegradation of MB dye [13,14].

There are several semiconductor materials that have been used for the promotion of photocatalytic activity. Among these, ecofriendly iron oxide (α-Fe_2_O_3_) nanomaterials exhibit favorable photocatalytic activity [15] due to their high specific surface area and high crystallinity, and they have the additional advantage of being low cost [16,17]. Hematite (α-Fe_2_O_3_) is the most stable iron oxide under ambient conditions, and due to its narrow band gap, it can act as a visible light photocatalyst [18]. This makes α-Fe_2_O_3_ a good potential candidate for a photocatalyst, yielding an enhancement of quantum efficiency when irradiated by visible light [9]. Nowadays, many researchers have tried to improve the photocatalytic behavior of Fe_2_O_3_ by coupling it with different semiconductor metal oxide nanoparticles, nanotubes, and nanoflowers [19,20]. Metal sulfide semiconductors [21], such as SnS_2_, usually have high light absorption qualities in the visible and shorter wavelengths near the infrared (IR) regions [22]. SnS_2_ has also been used as an n-type semiconductor due to its low band gap value of 2.2 eV [23]. Tin sulfide is also widely used as an energy material due to its simple binary composition and the abundance of tin and sulfur [24,25]. It is often used in research because of its unique physicochemical properties with low toxicity and good chemical stability and because it is inexpensive [26]. In addition, with its unique multilayer flower-like structure, SnS_2_ exhibits superior photocatalytic degradation of MB under visible light [27].

In this research, SiO_2_ nanoshells were used to maintain the structure of the nanocomposites and also to avert their aggregation during photocatalytic reactions [28,29]. This is very important in order to ensure the imperishability of the photocatalyst [30]. The preference of SiO_2_ for the inner core is based on the following two considerations: First, the silica nanospheres can produce multiple reflections of the visible light irradiated into the internal cavity [31]; second, the well-ordered and smooth structure of SiO_2_ promotes the adsorption of dye molecules to the semiconductor material [32,33]. Also, the SiO_2_ nanospheres are possessing large active sites that enable easy access of pollutants and, consequently, to improve the catalytic activity when the TiO_2_ and Fe_2_O_3_ nanoparticles are deposited on the SiO_2_ nanospheres [34,35,36].

We report a simplified approach for the synthesis of SnS_2_-SiO_2_@α-Fe_2_O_3_ nanocomposites by means of the hydrothermal method. The photocatalytic activity of the SnS_2_-SiO_2_@α-Fe_2_O_3_ nanocomposites was examined for the degradation of MB under irradiation by visible light. Additionally, samples with different weight percentages of SiO_2_@α-Fe_2_O_3_ on the SnS_2_ flowers were studied to find the optimal ratio for the best photocatalytic activity.

## 2. Experimental Procedure

### 2.1. Materials Used

Tin (IV) chloride pentahydrate (98%, Sigma Aldrich, St. Louis, MO, USA), Thioacetamide (TAA 99%, Acros, NJ, USA), Isopropyl Alcohol (Tedia, Fairfield, CT, USA), Ethanol (99%, Fisher Chemicals, NH, USA), tetraethylorthosilicate (TEOS 95%, Acros, NJ, USA), ammonia solution (Choneye Pure Chemicals, Miaoli, Taiwan), iron (III) chloride hexahydrate (97%, Sigma Aldrich, St. Louis, MO, USA), and MB were used. All the above reagents were analytical grade without any additional purification. Deionized water was used throughout the reaction and synthesis process.

### 2.2. Synthesis of Flower-Like SnS_2_

SnS_2_ flowers were prepared through a facile hydrothermal method using the same procedure as reported previously [37]. In brief, the procedure for the synthesis of the SnS_2_ flowers is as follows: 0.45 g SnCl_4_·5H_2_O and 0.33 g thioacetamide (TAA) were dissolved in 30 mL isopropyl alcohol. After 30 min of vigorous stirring, the solution was transferred to a Teflon-lined stainless-steel autoclave with a volume of 50 mL and then transferred to a muffle furnace where a hydrothermal was applied treatment at 180 °C for 24 h. The golden colored precipitates were then centrifuged and rinsed with copious amounts of deionized water and ethanol before being dried in a vacuum for 12 h at 80 °C.

### 2.3. Synthesis of SiO_2_@α-Fe_2_O_3_ Composite Spheres

In this experiment, silica spheres were synthesized using the same procedure described in our previous work [38]. A mixture of tetraethyl orthosilicate (TEOS) (5 mL), anhydrous ethanol (25 mL), and (5 mL) water was stirred at room temperature for 30 min, followed by the addition of 1 mL of ammonia solution. Next, 15 mL of anhydrous ethanol was added to the above mixture, which was stirred for 12 h at room temperature. After centrifugation, the SiO_2_ was collected and washed several times with water and ethanol. Finally, the obtained SiO_2_ spheres were dried in a vacuum oven at 60 °C for 12 h. Using ultrasonication, 100 mg of SiO_2_ spheres were uniformly dispersed in 40 mL of water, followed by the addition of 50 mg iron (III) chloride hexahydrate and 30 mg urea. The mixture was continuously stirred at 95 °C for 6 h. The FeOOH-coated SiO_2_ spheres were separated by centrifugation and then washed several times with water and ethanol. Finally, the spheres were dried in a vacuum oven at 60 °C for 12 h. The resulting FeOOH-coated SiO_2_ composite spheres were calcined at 450 °C for 4 h to get crystalline α-Fe_3_O_4_ nanoparticles.

### 2.4. Preparation of SnS_2_- SiO_2_@α-Fe_2_O_3_ Nanocomposites

The SnS_2_-SiO_2_@α-Fe_2_O_3_ (SSF) nanocomposites were prepared by adding 100 mg of flower-like SnS_2_ and different weight ratios (5, 10, 15, 20, 25 mg) of SiO_2_@α-Fe_2_O_3_ nanocomposites at 70 °C followed by effective stirring for 5 h. The resultant product obtained by centrifugation was then washed with ethanol and water. The obtained products were labeled SSF-X = 5, 10, 15, 20, 25 wt % corresponding to the weight ratio of the SiO_2_@α-Fe_2_O_3_ nanocomposites.

### 2.5. Degradation of MB Dye

The as-synthesized SnS_2_-SiO_2_@α-Fe_2_O_3_ (SSF-15 wt %) nanocomposites (40 mg) were dispersed in 100 mL of 5 ppm MB dye solution and stirred at 30 min in the dark. The reaction dye solution was then irradiated under visible light. A 410 nm LED light (Shenzhen Run lite Tech., Shenzhen, China; T2835-0.06W, 12V DC) was placed on the top of the sample bottle used as a visible light source. A 5 mL sample was taken every 15 min, and the degradation was monitored by using UV–Vis spectroscopy. The proceeding degradation of MB obtained by using α-Fe_2_O_3_, SnS_2_, and SiO_2_@α-Fe_2_O_3_ were observed to check on the photocatalytic activity.

### 2.6. Characterization

The X-Ray diffraction studies were carried out using the Cu-K_α_ line XRD (PANanalytical X’Pert PRO, Almelo, Netherlands), The FTIR experiment was carried out using (PerkinElmer Frontier FT-IR Spectrometer, Seer Green, UK) in the range between 400 and 4000 cm^−1^. The structure and morphology of the catalysts were analyzed using a field emission scanning electron microscopy (FE-SEM) (JEOL, JSM-7610F, Tokyo, Japan). The transmission electron microscopy (TEM) was carried out using (JEOL, JEM2100F, Peabody, MA, USA). The UV–Vis diffused reflectance spectra (DRS) were taken using an (Agilent Technology, Cary5000, Santa Clara, CA, USA). The surface area of the as-synthesized nanocomposites was obtained by Brunauer–Emmett–Teller (BET), using a (Micromeritics-Gemini V, ASAP 2020, Norcross, GA, USA). Photoluminescence (PL) studies were carried out using a (Dongwoo-Ramboss 500i, PL Spectrometer, Gyeonggi-Do, Korea). The X-ray photoelectron (XPS) spectroscopy was analyzed using a (JEOL, JPS-9030, Tokyo, Japan). The electrochemical impedance spectroscopy (EIS) was carried out using a Zive Potentiostat (SP100, eDAQ Inc., Colorado Springs, CO, USA).

## 3. Results and Discuss

### 3.1. X-Ray Diffraction

**Figure 1:**
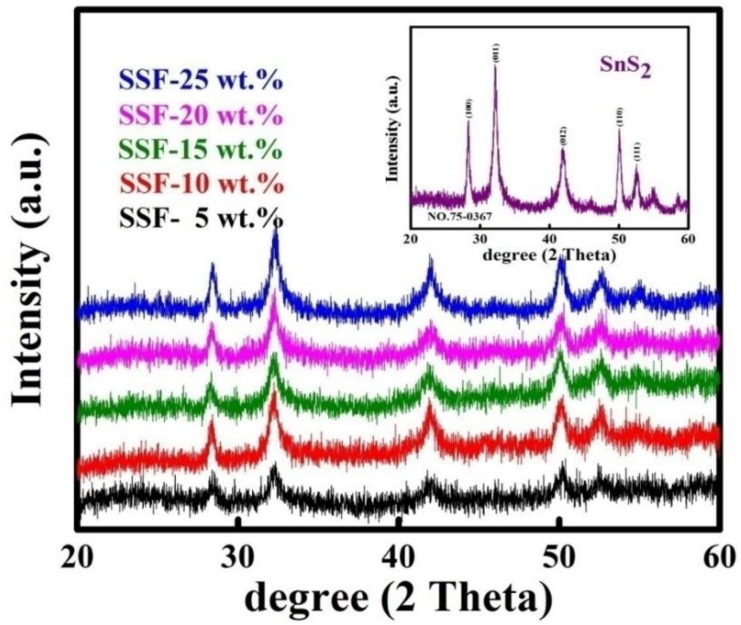
XRD patterns for SnS_2_-SiO_2_@α-Fe_2_O_3_ nanocomposites with different concentrations (SSF-5, 10, 15, 20, and 25 wt %). The inset shows the XRD pattern for as-synthesized flower-like SnS_2_.

The crystalline nature of the obtained samples was confirmed by XRD. The XRD results for the as-prepared SnS_2_ and SnS_2_-SiO_2_@α-Fe_2_O_3_ (SSF-X) (X = 5, 10, 15, 20, and 25 wt %) are shown in Figure 1. The inset to the figure shows the set of peaks at 28.29°, 32.2°, 41.8°, 50.1°, 52.5°, and 55.2°, which can be indexed to the pure hexagonal phase of SnS_2,_ as confirmed by JCPDS 75-0367 [39]. A peak at 23° confirms the amorphous SiO_2_, and the peak appearance at 33° and 35.8° corresponds to α-Fe_2_O_3_ in Figure S1. After the addition of various concentrations of SiO_2_@α-Fe_2_O_3_ to the SnS_2_ flowers, the intensity of the SnS_2_ decreased due to the deposition of the SiO_2_@α-Fe_2_O_3_ nanocomposites. Figure 1 clearly shows the 2θ peak at 32.2°, which corresponds to the (011) diffraction planes indexed to the (011) crystalline SnS_2,_ which in turn decreased with an increase in the concentration of the SiO_2_@α-Fe_2_O_3_ nanocomposites on SnS_2_, confirming the deposition of SiO_2_@α-Fe_2_O_3_ nanocomposites.

### 3.2. FE-SEM Structure

**Figure 2:**
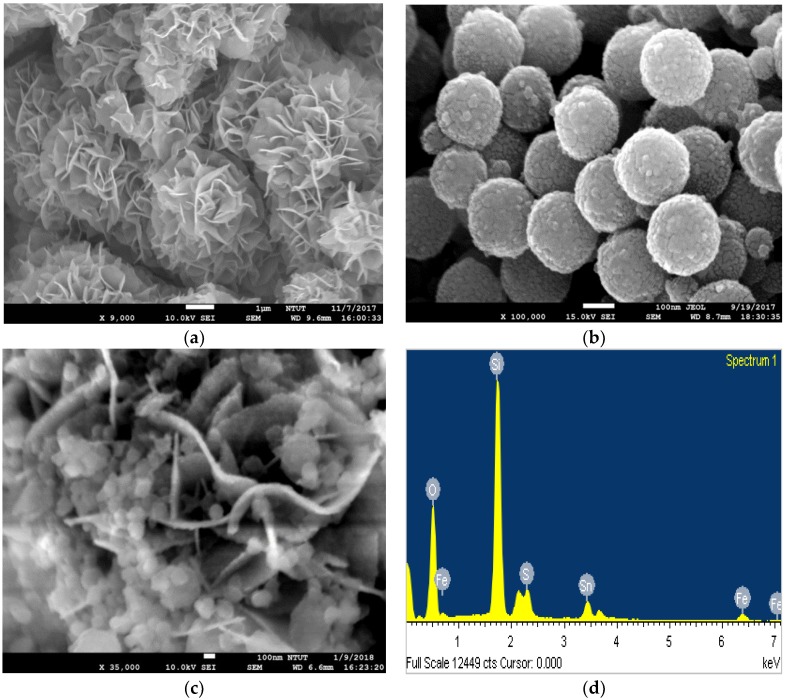
FE-SEM images of (**a**) Flower-like SnS_2_, (**b**) SiO_2_@α-Fe_2_O_3_ nanocomposites, (**c**) SnS_2_-SiO_2_@α-Fe_2_O_3_ nanocomposite, and (**d**) EDX spectra corresponding to a SnS_2_-SiO_2_@α-Fe_2_O_3_ nanocomposite.

The structural morphologies of SnS_2_, α-Fe_2_O_3_, and SnS_2_-SiO_2_@α-Fe_2_O_3_ were studied using FE-SEM. The flower-like morphology of the as-prepared SnS_2_ can be clearly observed in the FE-SEM images. This morphology is produced by the accumulation of several nanosheets assembled at one solitary point. Interestingly, the uniform nanosheet morphology is primarily caused by the intrinsic anisotropic growth of SnS_2_ crystals [37]. The size of each nanosheet is about 20 nm, and the size of the full flower-like structure is in a range between 3 and 4 µm, as shown in Figure 2a. In the SnS_2_-SiO_2_@α-Fe_2_O_3_ composites, α-Fe_2_O_3_ nanoparticles are deposited on the surface of SiO_2_ spheres with a size of around 150 nm, as shown in Figure 2b. The SiO_2_@α-Fe_2_O_3_ nanocomposites rest on the surface of the SnS_2_ flowers, as shown in Figure 2c. The SiO_2_ core-shell structure allows the multiple reflections of visible light, and the α-Fe_2_O_3_ and SnS_2_ are used to enhance the absorbance sites. An energy dispersive X-ray Analysis (EDX) observations confirmed the presence of SnS_2_-SiO_2_@α-Fe_2_O_3_ composites. The EDX results give energy peaks congruent with the elements of Sn, S, Fe, O, and Si in Figure 2d.

### 3.3. TEM

**Figure 3:**
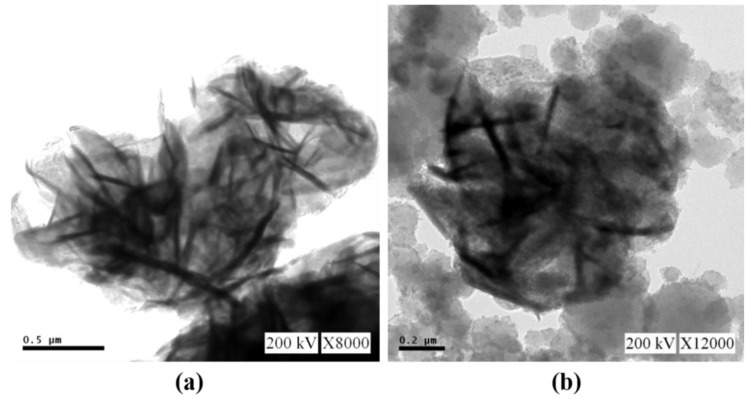
TEM images of (**a**) Flower-like SnS_2_, (**b**) SnS_2_-SiO_2_@α-Fe_2_O_3_ nanocomposite.

The detailed structural and morphological properties of as-prepared SnS_2_ and nanocomposites were examined by transmission electron microscopy (TEM). Figure 3 shows the TEM images of as-synthesized flower-like SnS_2_ (a). The SiO_2_@α-Fe_2_O_3_ nanospheres are arranged on the surface of SnS_2_ flowers (b). This clearly indicates that the SiO_2_@α-Fe_2_O_3_ nanocomposites are observed on the SnS_2_ flower, still maintains the flower like structure.

### 3.4. XPS

**Figure 4:**
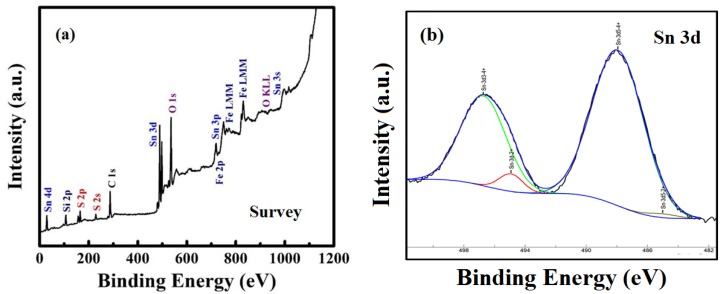

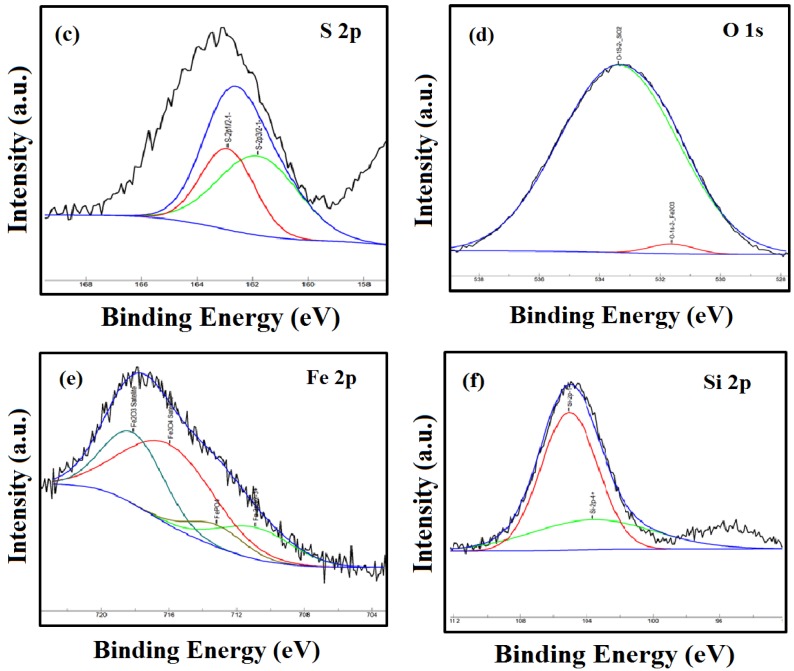
XPS (**a**) survey spectrum of SSF-15 wt % nanocomposite (**b**–**f**) Sn 3d, S 2p, O 1s, Fe 2p, and Si 2p.

To further confirm the elements, their composition, chemical bonding, and oxidation state of the materials, we used X-ray photoelectron spectroscopy (XPS). Figure 4a is the XPS survey spectrum of SnS_2_-SiO_2_@α-Fe_2_O_3_ (SSF-15 wt %) nanocomposite. This demonstrates that the corresponding signals of the main elements Sn, S, Si, O, and Fe were present in the composite. Figure 4b–f shows the high-resolution XPS spectra of Sn 3d, S 2p, O 1s, Fe 2p, and Si 2p. In Figure 4b, the distinctive peaks at 496.7 and 488 eV correspond to the binding energies of Sn 3d_5/2_ and Sn 3d_3/2_, respectively, indicating the Sn^4+^ oxidation state of Sn [40]. Figure 4c shows the enlarged XPS spectrum of S-contained peaks at 161.2 and 163.4 eV, ascribed to the binding energies of S 2p_3/2_ and S 2p_1/2_, showing the presence of S in S^2−^ state [41]. In the high-resolution XPS spectrum of O in Figure 4d, the typical peaks at 531.6 and 533.4 could be assigned to O 1s having O in O^2−^ state [42]. Figure 4e shows the narrow-scan XPS spectrum of Fe 2p having binding energies at 711.3, 715.9, and 718.3 eV, assigned to the Fe 2p_3/2_, satellite peak and Fe 2p_1/2_, respectively [43,44]. The peaks at binding energy 103.6 and 105.1 eV correspond to Si 2p_3/2_ and Si 2p_1/2_ of Si^4+^ in SiO_2_ [45]. From the XPS results, the successful formation of a SnS_2_-SiO_2_@α-Fe_2_O_3_ nanocomposite can be proved by the binding energies of each element.

### 3.5. EIS Spectra

**Figure 5:**
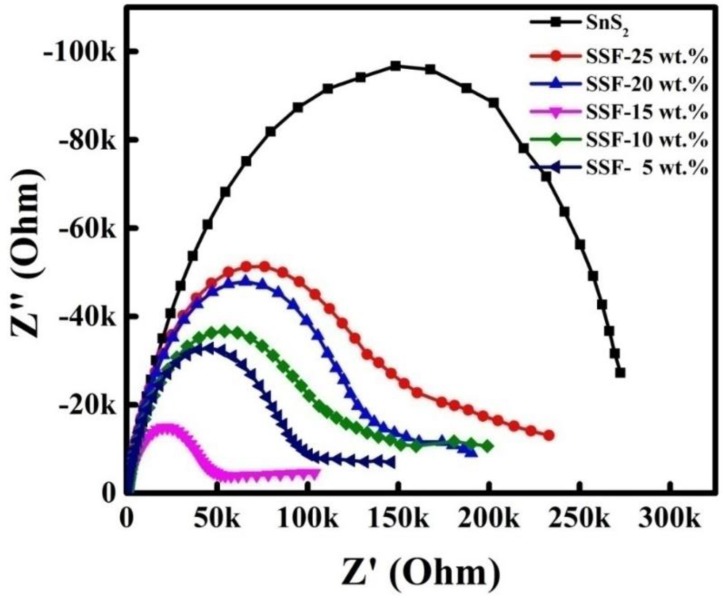
Electrochemical impedance spectroscopy (EIS) spectra of SnS_2_ and different concentrations of SSF-X nanocomposites (X = 5, 10, 15, 20, and 25 wt %).

The EIS spectra were obtained for the different SnS_2_-SiO_2_@α-Fe_2_O_3_ (SSF-X) (X = 5, 10, 15, 20, and 25 wt %) nanocomposite samples with a modified glassy carbon electrode (GCE) for the KCl (0.1 M) containing 5 mM of [Fe(CN)_6_]^3−^ and [Fe(CN)_6_]^4−^. This EIS spectrum shows the electrical and interfacial properties of the aforementioned nanocomposite materials. The SSF-15 wt % nanocomposite produces a smaller semi-circle in comparison to the other composites. In Figure 5, the smallest semi-circle, with a reduced diameter, indicates very low resistance. The electron transfer resistance observed is 154, 149, 150, 145, and 101 kΩ^−1^ for the various 25, 20, 15, 10, and 5 wt % composites, respectively. The low resistance observed for the SSF-15 wt % nanocomposite is due to the counterpart (stoichiometric) ratio between SnS_2_ and SiO_2_@α-Fe_2_O_3_. The deposition of SiO_2_@α-Fe_2_O_3_ on the SnS_2_ flowers plays an important role in enhancing the charge resistance, due to the low specific capacity of the nanocomposites. This increased electrical conductivity can also extend the lifespan of the charge carriers, as well as increase the reduction in the electron–hole recombination rate. It is also worth noting that higher impedance values were observed for the 15 wt % of SiO_2_@α-Fe_2_O_3_ nanocomposites, as opposed to all of the other nanocomposites.

### 3.6. UV–Vis DRS Spectra

**Figure 6:**
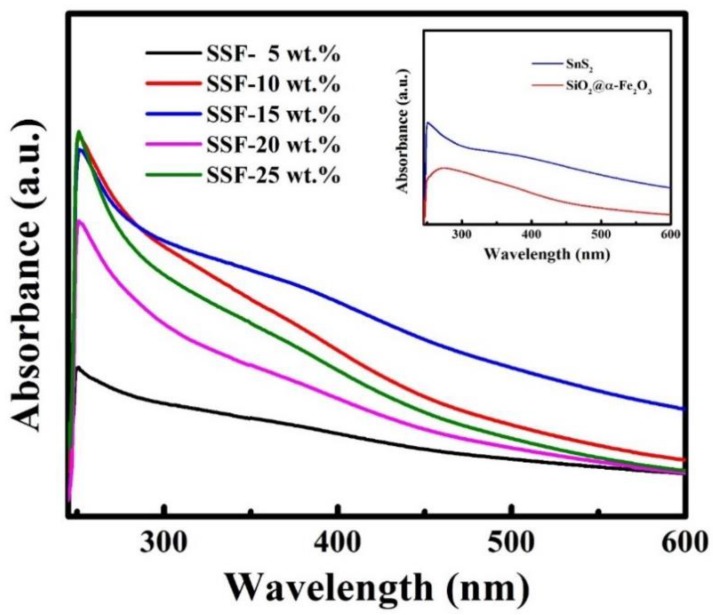
UV–Visible diffused reflectance spectra (DRS) spectra of as-synthesized SSF-X (X = 5, 10, 15, 20, 25 wt %); the inset shows DRS spectra of SnS_2_ and SiO_2_@α-Fe_2_O_3_.

The broad optical absorption capabilities in the entire visible light spectrum in Figure 6, which is commonly defined in the wavelength range of 400–700 nm, imply that all composites are efficient visible light-sensitive photocatalysts. The SSF-15 wt % indicates that composites have strong and broader absorption in the visible light region, suggesting their potential capabilities effectively utilize visible light energy. This can be attributed to the synergetic effect between SnS_2_ and α-Fe_2_O_3_ nanocomposites. Consequently, the composition SSF-15 wt % enhanced the absorption in the visible light region, which may lead to a higher photocatalytic activity when compared with other weight percentage compositions.

### 3.7. FTIR Spectra

**Figure 7:**
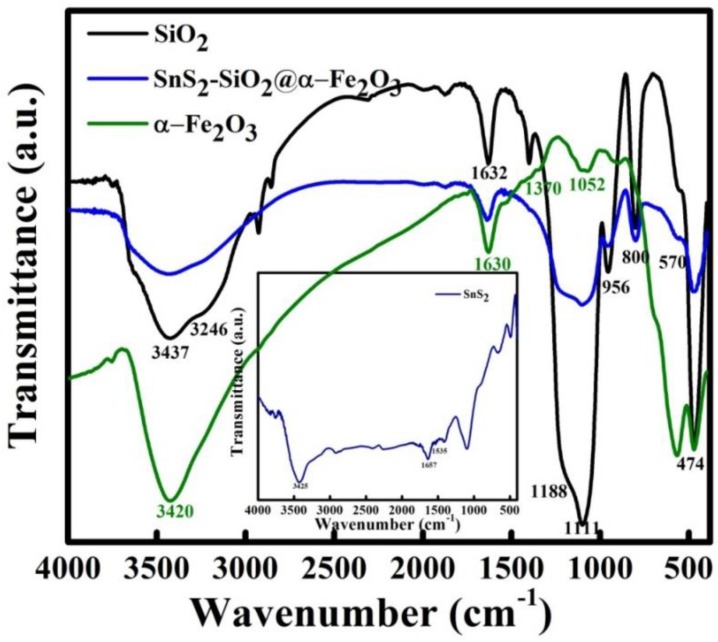
FTIR spectra of SiO_2_, α-Fe_2_O_3_, and SnS_2_-SiO_2_@α-Fe_2_O_3_ composites; inset shows the SnS_2_ spectra.

The FTIR spectra of the SnS_2_ flowers, SiO_2_ spheres, and α-Fe_2_O_3_ and SnS_2_-SiO_2_@α-Fe_2_O_3_ nanocomposites are shown in Figure 7. The inset to the figure shows the distinctive peaks at 1535 cm^−1^ and 1657 cm^−1^ representative of the SnS_2_. A strong stretching vibration of Si-O-Si at 1111 cm^−1^, a symmetric vibration of the Si-O-Si at 800 cm^−1^, and a symmetric stretching vibration of the Si-OH bond at 956 cm^−1^ and 3246 cm^−1^ can be observed. An absorption peak in the range between 3245 and 3450 cm^−1^ appears for all the samples after they have been annealed at 450 °C due to the stretching vibration of the OH band, which indicates the presence of the –OH group [38]. As the percentage of SiO_2_@α-Fe_2_O_3_ increases from low to high, there is not much variation in the FTIR spectra. However, the stretching vibrations, which are observed for all the samples, confirm the presence of OH groups. The bands observed at 474 cm^−1^ and 572 cm^−1^ are due to the Fe-O metal oxide stretching vibration modes, while the peak at 1052 cm^−1^ indicates the presence of the Fe-OH group.

### 3.8. Raman Spectroscopy

**Figure 8:**
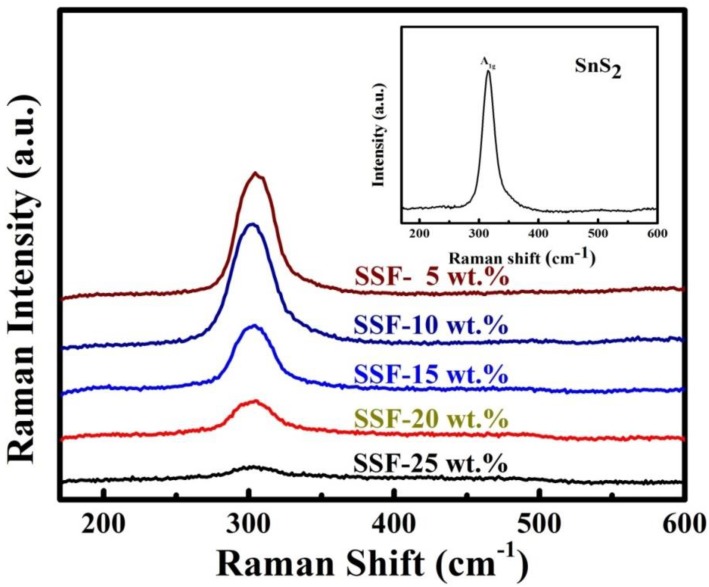
Raman spectra of SnS_2_-SiO_2_@α-Fe_2_O_3_ nanocomposites with various concentrations (SSF-5, 10, 15, 20, and 25 wt %); inset shows the Raman spectra of flower-like SnS_2_.

Figure 8 shows the Raman spectra for the SnS_2_-SiO_2_@α-Fe_2_O_3_ (SSF-X) (X = 5, 10, 15, 20, and 25 wt %) nanocomposites observed in the range of 100 to 1000 cm^−1^. The Raman spectrum for SnS_2_ exhibits an intense peak at about 315 cm^−1^, which is attributed to the A1_g_ mode [46,47] (Figure 8 [inset]). Increasing the concentration of SiO_2_@α-Fe_2_O_3_ produces no change in the SnS_2_ flower-like structure, as shown in Figure 8. For comparison, the Raman spectra of SiO_2_ and SiO_2_@α-Fe_2_O_3_ are shown in Figure S2. 

### 3.9. BET Surface

The surface areas of the as-synthesized SnS_2_-SiO_2_@α-Fe_2_O_3_ (SSF-X) (X = 5, 10, 15, 20, and 25 wt %) composites are characterized by nitrogen adsorption-desorption Brunauer–Emmett–Teller (BET) analysis, as shown in Table 1. From the results of BET analysis, the specific surface areas of SSF-X (X = 5, 10, 15, 20, and 25 wt %) are calculated to be 41.61, 39.41, 42.8, 41.93, and 40.47 m^2^/g, respectively. From the above results, it can be seen that the surface area of SSF-15 is slightly higher among all specimens. This implies that the enhancement of the adsorption capacity occurred in SSF-15, compared to that of the plain SnS_2_ flowers. 

### 3.10. PL Measurement

**Figure 9:**
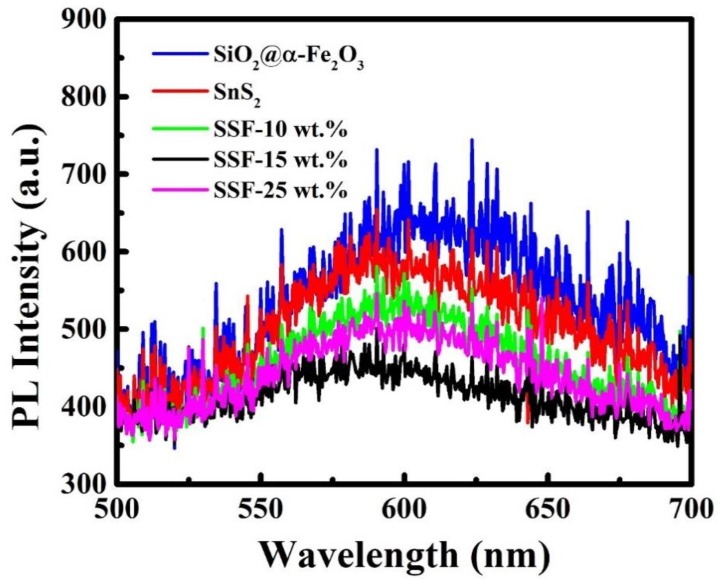
Photoluminescence spectra of SiO_2_@α-Fe_2_O_3_, SnS_2_, and SnS_2_-SiO_2_@α-Fe_2_O_3_ nanocomposites with different weight percentages (SSF-X) (X = 10, 15, and 25 wt %).

Figure 9, shows the PL spectra of SnS_2_-SiO_2_@α-Fe_2_O_3_ (SSF-X) (X = 10, 15, and 25 wt %) nanocomposites. The board peak at 600 nm indicates the emission band of SnS_2_ flower-like structure. After deposition of various concentrations of SiO_2_@α-Fe_2_O_3_ nanocomposites on SnS_2_, the peak at 600 nm is decreased, indicating the reduction of the recombination rate of electron–hole pairs. The SSF-15 wt % shows minimum intense PL spectra and demonstrates the efficient charge separation of the composites, as shown in Figure 9. This lower excitonic of PL intensity is due to the stronger capacity of dopants to capture photo-induced electrons, increase the separation rate of photoinduced electrons and holes, and increase photocatalytic activity. Further, a decrease in the light emission intensity indicates the direct radiative recombination and lower recombination of generated carriers. This result shows that a significant reduction in the recombination rate of photogenerated electron–hole pairs is observed in SSF-15 wt % when compared with other nanocomposites. The SSF-15 wt % composite has the lowest full width at half maximum (FWHM) value of 93.23 compared to that of SSF-10 wt % (99.61) and 25 wt % (105.67), respectively. The lowest FWHM value of SSF-15 wt % demonstrated the longer lifetime of the electron–hole pair and thus has the best intrinsic photocatalytic property.

### 3.11. Dye Degradation

**Figure 10:**
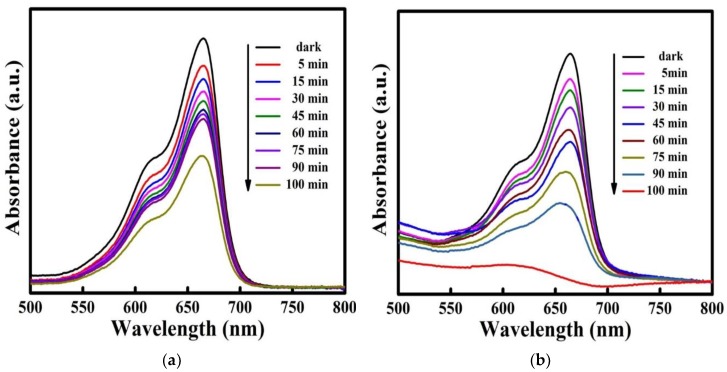
Photodegradation of MB dye in aqueous solution under visible light for (**a**) SnS_2_ and (**b**) SnS_2_-SiO_2_@α-Fe_2_O_3_ (SSF-15 wt %) nanocomposites.

Figure 10a,b show the process of MB dye degradation after exposure to visible light irradiation in the presence of SnS_2_ and SnS_2_-SiO_2_@α-Fe_2_O_3_ (SSF-15 wt %) photocatalysts. The UV–visible absorption spectrum shows a peak at 664 nm, as was observed for the MB dye in Figure 10a,b. When irradiation time was increased, the absorption intensity of the peak at 664 nm decreased, which confirms the progress of the degradation of the MB dye used as a synthesized photocatalyst. For the 15 wt % SnS_2_-SiO_2_@α-Fe_2_O_3_ sample, complete degradation of the MB dye took only 100 min. 

**Figure 11:**
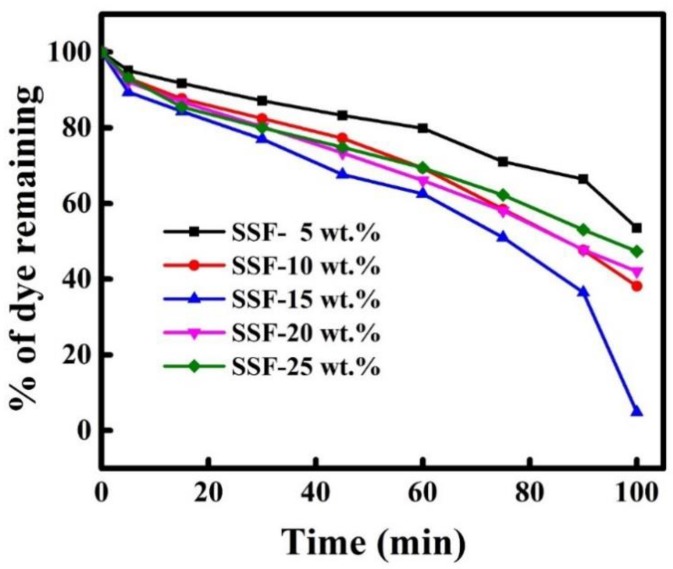
Catalytic activity of different SSF-wt % (SnS_2_-SiO_2_@α-Fe_2_O_3_) nanocomposites on the photodegradation of MB.

Furthermore, the photodegradation activities of SnS_2_-SiO_2_@α-Fe_2_O_3_ (SSF-X) with different concentrations of SiO_2_@α-Fe_2_O_3_ were analyzed. The degradation efficiency SSF catalyst with 5, 10, and 15 wt % SiO_2_@α-Fe_2_O_3_ increased as 47%, 62%, 96%, respectively. Upon increasing the concentration as 20 and 25 wt %, the degradation efficiency was decreased to 58% and 53%, respectively, as shown in Figure 11. It might be due to the accumulation of SiO_2_@α-Fe_2_O_3_ on SnS_2_ that reduces the penetration of light into the photocatalyst and generates the larger amount of photon adsorbed on the catalyst surface. Hence, the SSF-15 wt % of the SiO_2_@α-Fe_2_O_3_ is an appropriate amount for the excellent photodegradation of the MB solution. 

**Figure 12:**
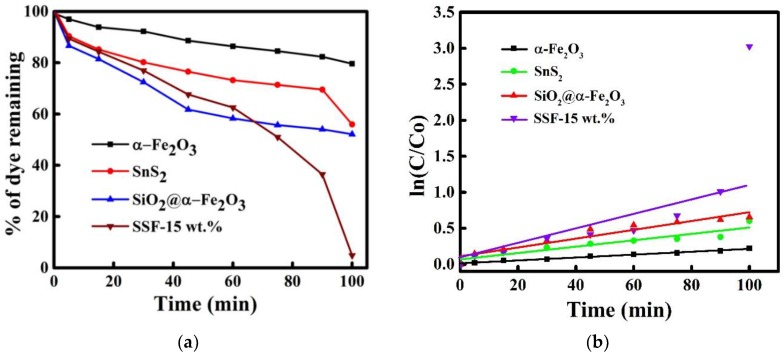

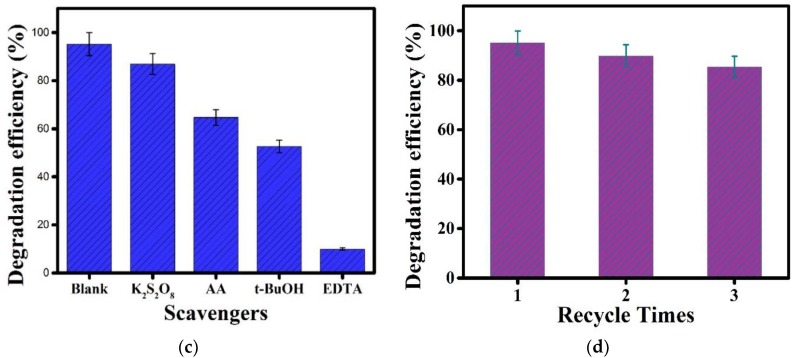
(**a**) Percentage of degradation and the remaining dye with respect to the different intervals of time (5 to 100 min) in the presence of the photocatalyst α-Fe_2_O_3_/SnS_2_/SiO_2_@α-Fe_2_O_3_/SnS_2_-SiO_2_@α-Fe_2_O_3_ under irradiation by visible light. (**b**) Plot of ln (C/C_o_) vs time. (**c**) Photodegradation efficiency of SSF-15 wt % in the presence of different active-species (scavengers). (**d**) Reusability of the SSF-15 wt % nanocomposite.

Figure 12a shows the percentage of MB dye remaining in the solutions for the SnS_2_, Fe_2_O_3_, SiO_2_@Fe_2_O_3_, and SnS_2_-SiO_2_@α-Fe_2_O_3_ (SSF-15 wt %) nanocomposites under visible light irradiation. Dye degradation efficiencies of 19%, 42%, 48%, and 96% were observed for the α-Fe_2_O_3_, SnS_2_, and SiO_2_@α-Fe_2_O_3_ and SnS_2_-SiO_2_@α-Fe_2_O_3_ (SSF-15 wt %) nanocomposites, respectively, at 100 min. The degradation of phenol (25 ppm) was also carried out in order to evaluate the catalytic activity of the SnS_2_-SiO_2_@α-Fe_2_O_3_ nanocomposite (Figure S3). In the presence of visible-light irradiation, the samples were taken at equal time intervals. The decrease in the distinct peak of phenol at 270 nm indicates the decomposition of phenol. This reveals the effective photocatalytic degradation of phenol done by the SnS_2_-SiO_2_@α-Fe_2_O_3_ nanocomposite. Therefore, the degradation of dye is related to the photocatalytic activity of SnS_2_-SiO_2_@α-Fe_2_O_3,_ rather than to the sensitization of dye.

The enhancement of the photocatalytic activity of the SnS_2_-SiO_2_@α-Fe_2_O_3_ (SSF-15 wt %) photocatalyst can be explained by two causes. First, the nanocomposites are prepared by a hydrothermal method having a higher surface area. Secondly, SSF-15 wt % has a very low impedance value in the EIS Nyquist plot. Here, the SiO_2_ spheres act as large adsorption sites and good locations for holding the α-Fe_2_O_3_ nanoparticles and adsorbing the dye molecules. During the photocatalytic reaction, the SnS_2_ and α-Fe_2_O_3_ act as photoactive centers and require a strong interaction between the dye and the surface of the catalyst for the effective degradation of dye [21,48]. Furthermore, the CB of α-Fe_2_O_3_ is slightly more negative, while the VB is much more positive than that of SnS_2_. This increases the driving force of holes migration when compared to electron transfer. This leads to a decline in the recombination of e−/h+ pairs, while the charge separation efficiency is increased. Therefore, an efficient charge separation may occur through the strong interfacial interaction in the SnS_2_/SiO_2_@α-Fe_2_O_3_ heterostructures, reducing the photogenerated charge recombination rate and improving the photocatalytic activity. The α-Fe_2_O_3_ nanoparticles used to trap electrons enhance the photocatalytic activity. Due to the reaction between these trapped electrons and dissolved oxygen in the system, the superoxide radical anions (O_2_^•−^) are formed, which in turn result in the formation of hydroxyl radicals (•OH). These •OH radicals are principally responsible for the oxidation of the organic compounds. This mechanism can be observed in terms of the active surface sites on the catalyst surface and the contact area of the dye and the catalyst. The 15 wt % concentration in SiO_2_@α-Fe_2_O_3_ nanocomposites increases the number of active sites, which in turn increases the number of hydroxyls (•OH) and superoxide (O_2_^•−^) radicals. Furthermore, degradation efficiency is reduced when the concentration of nanocomposites exceeds the normal range. This may be due to the agglomeration of the nanocomposites, which reduces the penetration of light, as well as the absorbance of the dye and the catalyst.

In Figure 12b, the plot of ln(C/Co) vs time shows that the photocatalytic degradation of MB follows the pseudo-first-order kinetics. The rate constant-k value for the SnS_2_-SiO_2_@α-Fe_2_O_3_ composite is 1.96 × 10^−2^ min^−1^, which is about 8, 4, and 3 times greater than that for α-Fe_2_O_3_ (2.01 × 10^−3^ min^−1^), SnS_2_ (4.43 × 10^−3^ min^−1^), and SiO_2_@α-Fe_2_O_3_ (6.12 × 10^−3^ min^−1^). The sudden increase in rate constant (k) between 90 and 100 min was due to the gradual rise in pH of the reaction medium (7.1–10.3). The oxidation potential, physicochemical, and surface charge properties of the catalyst were affected by the pH of the solution. The alkaline medium favored (i.e., the formation of •OH by the influences of OH^−^ ions on the surface of the catalyst) the decolorization of the dye more than the acidic medium [49,50]. The highest photocatalytic activity was attained with the SSF-15 wt % nanocomposite. Hence, we can prove that the as-synthesized nanocomposite heterostructures enhance the photocatalytic activity. In photocatalytic oxidation reactions, there are several reactive species, such as •OH, O_2_^•−^, holes, and electrons involved in the reaction. To examine the contribution of these species, tertiary butyl alcohol (t-BuOH), potassium persulfate (K_2_S_2_O_8_), acrylamide (AA), and ethylenediaminetetraacetic acid (EDTA) were used as scavenger materials for •OH, electrons, O_2_^•−^, and the holes, respectively, in the reaction process (Figure 12c). The degradation efficiency slightly decreased when the addition of K_2_S_2_O_8_ showed that the electrons play a minimal role in the photocatalytic reaction. The degradation rate of MB further decreased with the addition of AA, indicating reduced involvement of the O_2_^•−^ species. The degradation efficiency reduced during the addition of t-BuOH, which shows the partial involvement of •OH. The rate of MB degradation was significantly affected by EDTA, clearly indicating that holes play a major role in the photocatalytic degradation reaction.

To prove the stability and durability of the SnS_2_-SiO_2_@α-Fe_2_O_3_ nanocomposite photocatalyst, the recycle experiments were carried out (Figure 12d). Moreover, the results show that only 9.75% of degradation efficiency was lost during the third cycle, which proves that the SnS_2_-SiO_2_@α-Fe_2_O_3_ nanocomposites possessed higher stability and reusability efficiency (Figure S4).

## 4. Conclusions

Flower-like SnS_2_ and SnS_2_-SiO_2_@α-Fe_2_O_3_ nanocomposites with different weight percentages were successfully synthesized using a simple hydrothermal method. The SSF-15 wt % nanocomposites have higher catalytic activity than other samples. The as-prepared nanocomposites enhanced the amount of adsorption of cationic dye (MB) molecules with the addition of SiO_2_@α-Fe_2_O_3_ composites on the SnS_2_ flowers, thereby increasing the photocatalytic degradation activity. Due to the simplicity of preparation, it is possible to produce a low-cost photocatalyst with higher activity and stability, making it a good candidate material for applications in environmental purification. Based on our results, it should be noted that •OH, O_2_^•−^, and the holes played a vital role and enhanced the photocatalytic reaction.

## Figures and Tables

**Table 1:** Brunauer–Emmett–Teller (BET) surface areas of various SnS_2_-SiO2@α-Fe2O3 (SSF-X) (X = 5, 10, 15, 20, and 25 wt %) nanocomposites.

Sample	BET Surface Area
SnS_2_ flowers	41.88 m^2^/g
SSF-5 wt %	41.61 m^2^/g
SSF-10 wt %	39.41 m^2^/g
SSF-15 wt %	42.82 m^2^/g
SSF-20 wt %	41.93 m^2^/g
SSF-25 wt %	42.47 m^2^/g

## Supplementary Materials

The following are available online at http://www.mdpi.com/1996-1944/11/6/1030/s1, Figure S1: XRD patterns for SiO_2_ and SiO_2_@α-Fe_2_O_3_, Figure S2: Raman spectra of SiO_2_ and SiO_2_@ α-Fe_2_O_3_, Figure S3: UV-Vis absorbance spectra of phenol in the presence of SnS_2_-SiO_2_@α-Fe_2_O_3_, Figure S4: Comparison of XRD patterns of SSF-15 wt % with the same sample after three cycles.
