# Supplementary material for: Degradation of Methylene Blue Dye in the Presence of Visible Light Using SiO2@α-Fe2O3 Nanocomposites Deposited on SnS2 Flowers
Source: Materials (Basel). 2018 Jun 17;11(6):1030. doi: 10.3390/ma11061030 (PMC6025432; doi:10.3390/ma11061030)
Supplement: Supplementary file 1 [file materials-11-01030-s001.pdf]

Supplementary Materials

Degradation of Methylene Blue Dye in the Presence of Visible Light Using SiO_2@α-Fe_2O_3 Nanocomposites Deposited on SnS_2 Flowers

Sridharan Balu [1], Kasimayan Uma [2,*], Guan-Ting Pan [1], Thomas C.-K. Yang [1,2,*] and Sayee Kannan Ramaraj [3]

1 Department of Chemical Engineering and biotechnology, National Taipei University of Technology, Taipei 106, Taiwan, bsridharanbsc.12@gmail.com (S.B.); t6679013@gmail.com (G.-T.P.)
2 Precision Analysis and Research Center, National Taipei University of Technology, Taipei 106, Taiwan
3 PG & Research Department of Chemistry, Thiagarajar College, Madurai 625009, Tamilnadu, India; sayeekannanramaraj@gmail.com
* Correspondence: umamahesh16@gmail.com (K.U.); ckyang@mail.ntut.edu.tw (T.C.-K.Y.)

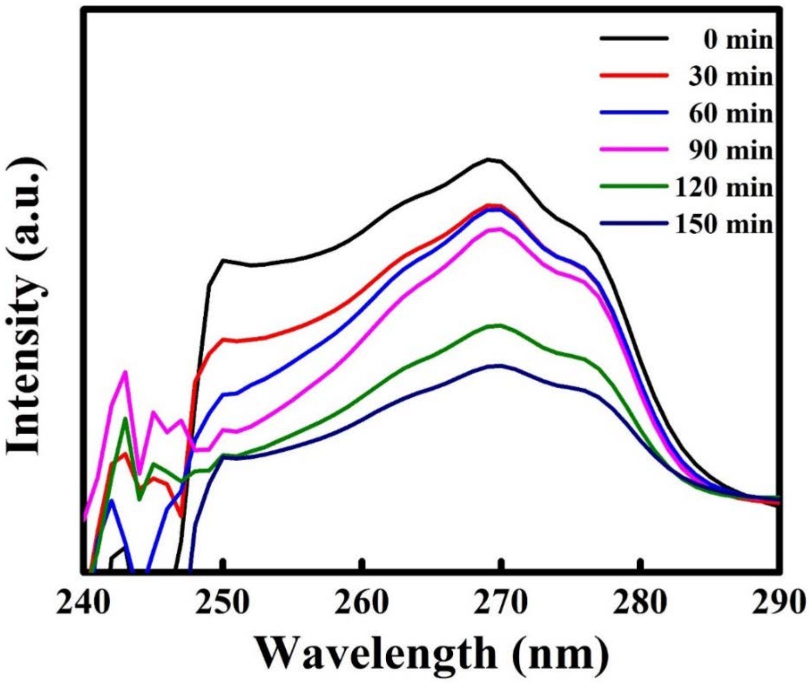

Figure S1. UV-Vis absorbance spectra of phenol in the presence of SnS_2-SiO_2@α-Fe_2O_3 (SSF-15 wt %) nanocomposite.

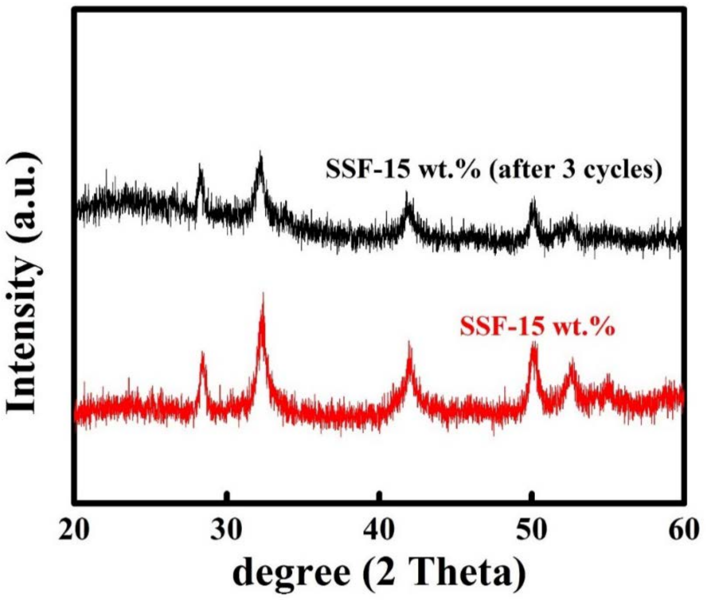

Figure S2. Comparison of XRD patterns of SSF-15 wt % with the same sample after three cycles

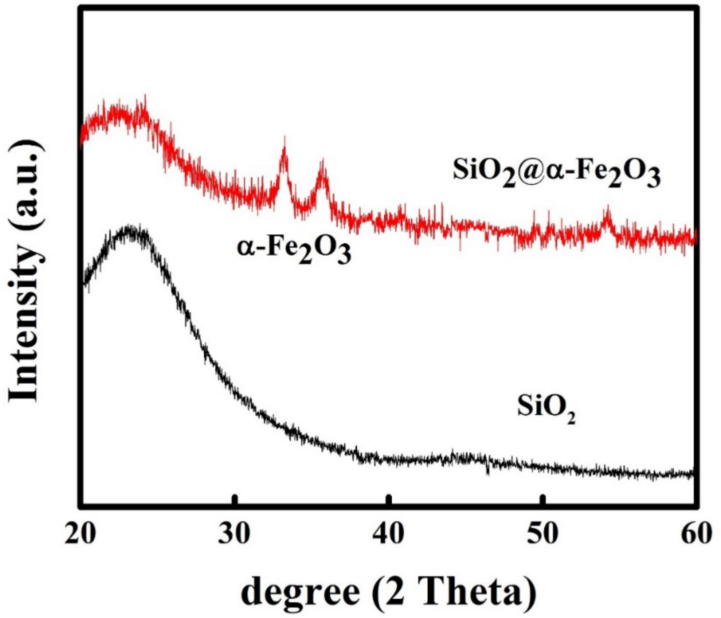

Figure S3. XRD patterns for SiO2 and SiO2@ α-Fe2O3

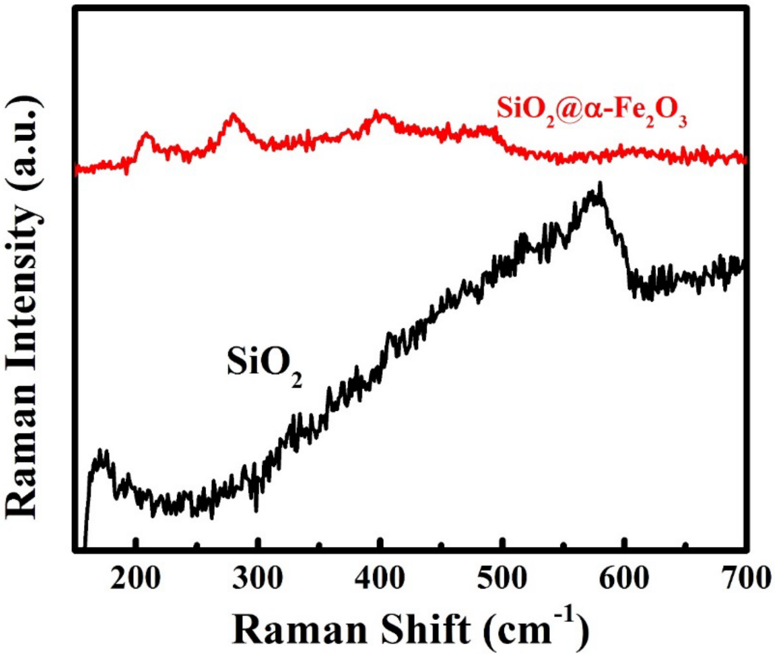

Figure S4. Raman spectra of SiO2 and SiO2@ α-Fe2O3
